# CUTS RNA Biosensor for the Real-Time Detection of TDP-43 Loss-of-Function

**DOI:** 10.1101/2024.07.12.603231

**Published:** 2024-07-12

**Authors:** Longxin Xie, Jessica Merjane, Cristian A Bergmann, Jiazhen Xu, Bryan Hurtle, Christopher J Donnelly

**Affiliations:** 1 Department of Neurobiology, University of Pittsburgh School of Medicine, Pittsburgh, PA, USA; 2 School of Medicine, Tsinghua University, China; 3 LiveLikeLou Center for ALS Research, University of Pittsburgh School of Medicine, Pittsburgh, PA, USA; 4 Interdisciplinary Biomedical Graduate Program Cellular and Molecular Pathology, University of Pittsburgh, Pittsburgh, PA, USA; 5 Center for Neuroscience at the University of Pittsburgh, Pittsburgh, PA, USA; 6 Pittsburgh Institute for Neurodegeneration, University of Pittsburgh, Pittsburgh, PA, USA; 7 Center for Protein Conformational Diseases, University of Pittsburgh, Pittsburgh, PA, USA

## Abstract

Given the mounting evidence implicating TDP-43 dysfunction in several neurodegenerative diseases, there is a pressing need to establish accessible tools to sense and quantify TDP-43 loss-of-function (LOF). These tools are crucial for assessing potential disease contributors and exploring therapeutic candidates in TDP-43 proteinopathies. Here, we develop a sensitive and accurate real-time sensor for TDP-43 LOF: the CUTS (CFTR UNC13A TDP-43 Loss-of-Function) system. This system combines previously reported cryptic exons regulated by TDP-43 with a reporter, enabling the tracking of TDP-43 LOF through live microscopy and RNA/protein-based assays. We demonstrate CUTS’ effectiveness in detecting LOF caused by TDP-43 mislocalization and RNA binding dysfunction, and pathological aggregation. Our results highlight the sensitivity and accuracy of the CUTS system in detecting and quantifying TDP-43 LOF, opening avenues to explore unknown TDP-43 interactions that regulate its function. In addition, by replacing the fluorescent tag in the CUTS system with the coding sequence for TDP-43, we show significant recovery of its function under TDP-43 LOF conditions, highlighting CUTS’ potential for self-regulating gene therapy applications. In summary, CUTS represents a versatile platform for evaluating TDP-43 LOF in real-time and advancing gene-replacement therapies in neurodegenerative diseases associated with TDP-43 dysfunction.

## Introduction

Amyotrophic lateral sclerosis (ALS) is a progressive and fatal neurodegenerative disease (NND) characterized by a persistent degeneration of the neurons of the spinal cord and motor cortex (Feldman et al., 2022). The dysregulation of the RNA binding protein (RBP), TAR DNA-binding protein 43 (TDP-43) is a hallmark pathobiology observed in ~97% of all ALS patients that (Arai et al., 2006; Neumann et al., 2006) ~45% of Frontotemporal lobar degeneration (FTLD) patients (S. C. Ling et al., 2013; Neumann et al., 2006), and 40%−60% of Limbic Associated TDP-43 Encephalopathy (LATE) patients (McKee et al., 2010; Nelson et al., 2019; Tremblay et al., 2011). Under physiological conditions, TDP-43 orchestrates many cellular processes critical for neuronal health and homeostasis, including regulating RNA metabolism, splicing, and stress response pathways. However, in disease, TDP-43 is depleted from the nucleus and mislocalizes to the cytoplasmic compartment, losing the ability to perform its canonical functions and transitioning into insoluble aggregates (Hurtle et al., 2023).

Recent efforts increasingly focus on the implications of TDP-43’s loss of splicing function and its consequence on disease onset and progression (Brown et al., 2022; Buratti et al., 2001; Klim et al., 2019; J. P. Ling et al., 2015; Ma et al., 2022; Melamed et al., 2019). Physiologically, TDP-43 selectively binds to specific UG-rich sequences within pre-mRNA transcripts, providing precise control over the alternative splicing of a subset of conserved targets, thereby modulating their gene expression and cellular function (Kuo et al., 2009; Lukavsky et al., 2013). This regulatory function is key in repressing the incorporation of TDP-43-mediated aberrant ‘cryptic exons’ (CE), non-conserved intronic regions whose inclusion has been linked to cellular toxicity and pathological consequences (Brown et al., 2022; Klim et al., 2019; J. P. Ling et al., 2015; Ma et al., 2022; Mehta et al., 2023; Melamed et al., 2019; Polymenidou et al., 2011). Noteworthy examples of transcripts susceptible to aberrant CEs in the absence of functional TDP-43 include *STMN2* (Klim et al., 2019; Melamed et al., 2019) and *UNC13A* (Brown et al., 2022; Ma et al., 2022), both pivotal in maintaining the integrity and physiological function of neurons, such as axon regeneration and motor neuron firing (Shin et al., 2012, 2014; Varoqueaux et al., 2002; Willemse et al., 2023). Therefore, their aberrant splicing patterns and loss-of-function (LOF) in disease highlights the critical role of TDP-43 in preserving neuronal health and function through the regulation of key neuronal health.

A well-established and currently available method to accurately monitor and quantify TDP-43’s splicing function has frequently relied on employing cryptic exon 9 inclusion in the human cystic fibrosis transmembrane conductance regulator (CFTR) transgene (Buratti et al. 2001; Ayala et al. 2006). However, this approach can only be used at the RNA level, limiting its functionality as a reporting system for high-throughput screens or *in vivo* applications. Furthermore, endogenous cryptic exons exhibit variable responses to TDP-43 loss, may be cell type-specific, and are still being defined. To address this, we designed a screening tool, the ‘CFTR-UNC13A TDP-43 loss-of-function Sensor’ (CUTS) system, engineered to detect TDP-43 LOF and output a proportional and quantifiable GFP signal or other reporters. By combining previously described TDP-43 binding targets, we significantly improved the sensitivity measurable in real-time by standard assays. Physiological TDP-43 promotes the splicing of CUTS CE, resulting in a frameshift and early stop codon that prevents GFP expression. TDP-43 LOF activates the CUTS biosensor by maintaining the CE inclusion and allowing GFP to express. Here, we highlight the CUTS system sensitivity in response to TDP-43 loss. The CUTS design allows for GFP expression with direct proportionality to TDP-43 LOF, enabling the detection of changes in TDP-43 function, even when alterations in TDP-43 itself cannot be directly measured by traditional means, including qPCR, western blot, and immunofluorescent imaging.

In this study, we highlight the utility and sensitivity of the CUTS biosensor. We also show that aberrant TDP-43 phase transitions or mislocalization disrupt TDP-43 splicing function via expression of well-established RNA binding deficient and cytoplasmic TDP-43 constructs. Lastly, we replaced the GFP cassette with the wildtype *TARDBP* coding sequence (CUTS-TDP43) to highlight the utility of CUTS biosensor as a regulator of a payload in response to TDP-43 loss of function implicating its potential use in safeguarded gene replacement therapies associated with TDP-43 LOF. The CUTS biosensor presented here represents a significant technological advancement in available TDP-43 biosensors and offers a reliable and highly sensitive means to dynamically monitor TDP-43 function. Implementing CUTS may facilitate the rapid screening of potential therapeutics to restore TDP-43 functionality, monitor TDP-43 function to understand disease-associated dysfunction, or deliver a genetic payload in response to TDP-43 loss.

## Results

### CUTS TDP-43 LOF sensor design utilizing known Cryptic Exons

**Figure 1:**
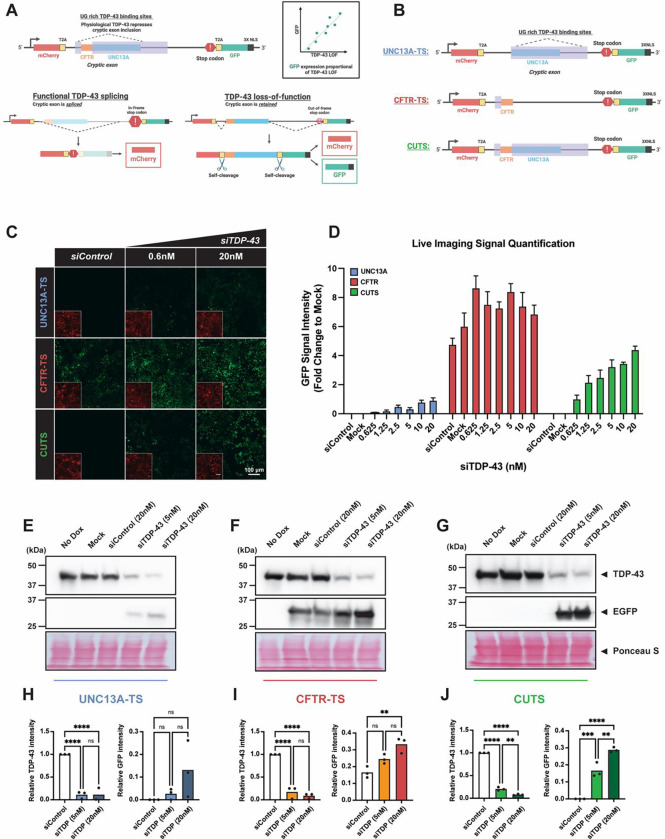
CUTS is more precise than UNC13A-TS and CFTR-TS as a TDP-43 splicing sensor. Comparison of stable polyclonal HEK cells expressing UNC13A-TS, CFTR-TS, or CUTS following treatment with siRNA control (siControl) (20nM) or TDP-43 (siTDP-43) (0.6nM - 20nM). Cells were reverse transfected with siRNA treatment in complete media supplemented with doxycycline (1000 ng/mL). After 72 h, cells were analyzed by live imagining and protein lysate was harvested for western blot analysis. **(A)** Schematic of the TDP-43 loss of function Sensor (TS) system design. **(B)** Overview of the UNC13A-TS, CFTR-TS, and CUTS cassette design. **(C)** Representative live imaging of TS comparison (10X). **(D)** Mean intensity quantification of GFP signal intensity as shown in (C). **(E-G)** Western blot analysis of (E) UNC13A-TS, (F) CFTR-TS, and (G) CUTS developing again GFP and TDP-43 proteins. **(H-J)** Relative pixel quantification of GFP and TDP-43 band normalized to total protein (Ponceau S) for the indicated TS from E-G. Statistical significance was determined by one-way ANOVA and Tukey’s multiple comparison test (* = P < 0.03; ** = P < 0.002; *** = P < 0.0002; **** = P < 0.0001). Green = GFP signal; red = mCherry signal. Scale bar = 100 μm. N=3 biological replicates.

To improve the detection of TDP-43 LOF, we designed a novel TDP-43 LOF sensor (TS) for real-time screening-based detection using previously reported genes known to undergo TDP-43 regulated splicing (*UNC13A* and *CFTR*). The TS cassettes are constructed with a constitutively expressed mCherry, followed by a TDP-43 regulated CE and a GFP linked to a 3x nuclear localization signal (NLS), each separated by a T2A self-cleavage sequence (Figure 1A). We positioned the GFP reporter outside the mCherry open reading frame (ORF), introducing an early stop codon upstream to the GFP (Figure 1A). This strategic design achieved three key outcomes: (1) Under physiological TDP-43 levels, the binding of TDP-43 to the CE and UG-rich sequence should promote complete intronic splicing, maintaining the in-frame stop codon and allowing only mCherry expression. (2) TDP-43 loss and/or failure to bind the CE sites, will result in CE retention and a subsequent frameshift, causing resulting in an out-of-frame stop-codon GFP codon inclusion. (3) The GFP output of the TS should be proportional to the level of TDP-43’s LOF (Figure 1A).

### CUTS is more precise than CFTR and UNC13A as a TDP-43 LOF biosensor

To assess the functionality of TS, we generated CFTR-TS and UNC13A-TS, which utilized the TDP-43 regulated CEs from *CFTR* and *UNC13A*, respectively (Figure 1B). Additionally, we engineered a combined construct termed CUTS (CFTR-UNC13A TS), integrating both CE sequences (Figure 1B). Additional base modifications were incorporated into the cassette designs to prevent unexpected stop codons within the CE regions (see Table S1 for full sequence details). Each construct was coupled with a Tet3g promotor, cloned into a Piggybac vector, and stably expressed in HEK293 cells. To evaluate the accuracy and reliability of the three TS constructs, we performed a TDP-43 LOF assay using increasing siRNA concentrations, followed by live confocal imaging and western blot (WB) analysis (Figure 1 and S1). Across all cell lines, we observed constitutive mCherry expression and an increased trend in GFP signals with higher siTDP43 concentrations. The CFTR-TS construct exhibited a notably high baseline, with detectable GFP leakage in control groups without TDP-43 loss (Figure 1C–1D, 1F, 1I, S1). In contrast, the UNC13A-TS construct demonstrated superior accuracy compared to CFTR-TS, showing no detectable GFP expression under control conditions in both imaging-based and WB analyses (Figure 1C–1E, 1H, S1). However, the UNC13A-TS showed limited sensitivity, with only a modest GFP signal (17% of CUTS) under high quantities of siTDP43 treatment. Interestingly, cells expressing the CUTS construct exhibited a synergistic effect from both the CFTR and UNC13A CE sequences, achieving high sensitivity evidenced by a clear dose-responsive GFP expression with increasing siTDP43 concentrations, while maintaining high accuracy with minimal leakage via imaging and GFP immunoblotting (Figure 1C–1D, 1G, 1J, S1A–S1B). Given the promising accuracy of the CUTS sensor, we proceeded to further characterize the CUTS RNA biosensor.

### CUTS demonstrates ultra-sensitivity in detecting low-level TDP-43 loss-of-function

**Figure 2:**
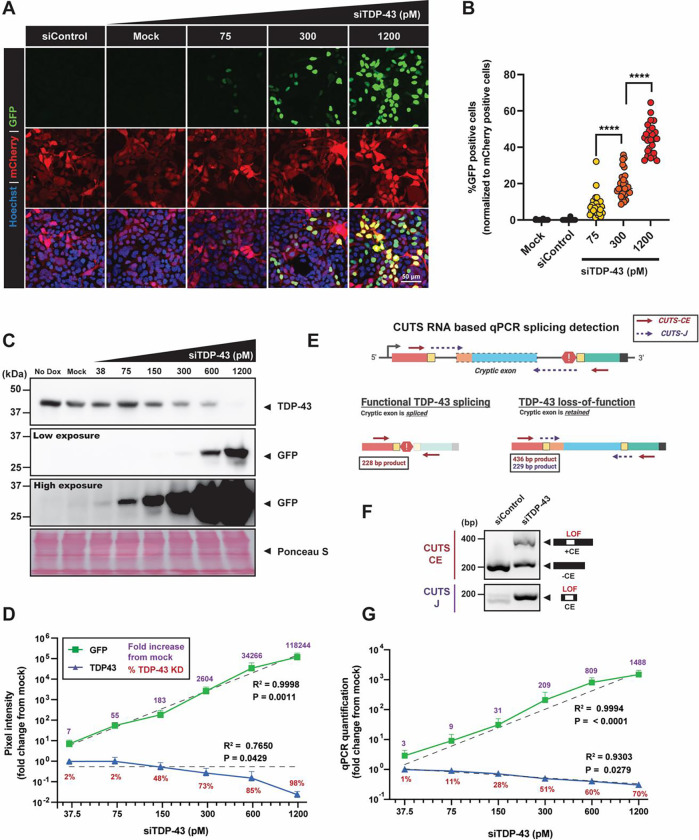
CUTS enables detection of TDP-43 loss-of-function with ultra-low siRNA treament. Low-dose siRNA TDP-43 (siTDP-43) treatment was performed in stable polyclonal HEK cells expressing CUTS. CUTS-expressing cells were reverse transfected with siRNA control (siControl) or siTDP43 in a dose-response curve (38 to 1200pM) in doxycycline supplemented media (1000ng/ml) for 72hr. (A) Representative immunofluorescence images of CUTS-expressing HEK cells under low doses of siRNA TDP-43 treatment. (60X). (B) Mean intensity quantification of GFP signal from (A) with normalization to the number mCherry positive cells. (C) Western blot of GFP and TDP-43 proteins from HEK cell lysate expressing CUTS under low doses of siRNA TDP-43. Ponceau S is shown as a loading control. (D) Pixel intensity quantification of the GFP and TDP-43 bands shown in (C), presented as fold-change from the mock-treated sample. (E) Schematic showing the position of qPCR primers, developed to detect CUTS cryptic exon inclusion (referred to as ‘CUTS-CE’ and ‘CUTS-J’). (F) Representative agarose gel showing qPCR product from melting curve detecting CUTS cryptic exon inclusion using the primers shown in (E). (G) qPCR quantification of the siTDP-43 dose curve presented as fold-change from the mock-treated sample. Purple text indicates the GFP fold change from the mock-treated sample. Red text indicates the percentage of total detectable TDP-43 knockdown. Linear regression analysis shown in (D) and (G) was performed on Log values. Fitting method = least squares regression. Green = GFP; red = mCherry. Scale bar = 50 μm. N=3 biological replicates.

To challenge the stability and sensitivity of CUTS, we next conducted an ultra-low dose TDP-43 siRNA transfection, ranging from 38 – 1200 pM. Immunofluorescence staining (IF) revealed a consistent increase in both GFP intensity and GFP-positive cell ratios in CUTS-expressing cells in response to elevated siTDP43, with minimal baseline expression observed (Figure 2A–2B). We confirmed the ultra-sensitivity and accuracy of CUTS using WB analysis, demonstrating measurable GFP even at the lowest doses of siTDP43 assessed. While changes in TDP-43 levels were undetectable by WB at siTDP43 doses of 37.5 – 75 pM (measured as 2% TDP-43 KD by WB), the CUTS system demonstrated a 7 to 55-fold increase in GFP expression at these doses compared to baseline (Figure 2C–2D). This increase in GFP expression continued consistently up to the highest dose of siTDP43 (1,200 pM; 98% TDP-43 KD), showcasing a 118,224-fold increase in GFP compared to baseline. Pearson’s correlation analysis confirmed a highly significant relationship between GFP expression and siTDP43 dose (P = 0.0011), while the correlation between measurable TDP-43 and siTDP43 concentration was less significant (P = 0.0429). Linear regression analysis between the logarithmic GFP fold increase and siRNA doses demonstrated an exceptional linear relationship (R^2^ = 0.9998). These results indicate that GFP expression produced by CUTS is a more sensitive method for detecting TDP-43 KD (and therefore LOF) than TDP-43 WB detection. The linear relationship between GFP and siTDP-43 dose also demonstrates the potential of CUTS to be used as a predictive model for TDP-43 LOF.

We assessed CUTS’ sensitivity at the transcript level using the siTDP43 dose curve and RT-qPCR assessment in Figure 2C–D. To determine the relative amount of CUTS’ CE retention, we designed primers targeting either the entire transcript or the specific junction sites of the CE (Figure 2E–2F). The RT-qPCR quantification demonstrated increased sensitivity at detecting changes in TDP-43 levels compared to WB analysis, with the capability of detecting changes in TDP-43 between each siTDP43 dose (1 – 70% TDP-43 KD) in a linear manner (R^2^ = 0.9303). Using CUTS detection, we observed a clear linear logarithmic relationship between the amount of CUTS CE-retention and the increasing siTDP43 doses (R^2^=0.9994). Even at 1% KD in TDP-43, the CUTS system detected a 3-fold increase in CE retention compared to baseline, which increased to a 1,488-fold increase at 70% TDP-43 KD (Figure 2G). As with our WB analysis (Figure 2D), there was a significant correlation between both GFP expression and TDP-43 when correlated to siTDP43 dose (P < 0.0001 and P = 0.0279, respectively). Thus, these data indicate that CUTS is a reliable approach to quantifying TDP-43’s loss across an extensive range, highlighting its ability as a TDP-43 LOF biosensor. Additionally, CUTS demonstrated ultra-sensitivity under low-level TDP-43 KD, beyond the detection limit of both WB and RT-qPCR.

### Pathological TDP-43 phase transitions or mislocalization activate the CUTS biosensor

In ALS/FTLD, the absolute TDP-43 level remains largely unaffected. Instead, TDP-43 undergoes pathological mislocalization and/or phase transitions likely due to a reduction in RNA binding, which reduces the functional cellular TDP-43. To evaluate whether these events contribute to TDP-43 loss-of-function, we tested CUTS’s ability to detect TDP-43 LOF caused by TDP-43 mislocalization or aggregation via aberrant phase transitions. We transfected CUTS HEK293 cells with four tagged TDP-43 isoforms: (1) TDP-43^WT^, (2) TDP-43^cyto^, (3) TDP-43^5FL^, and (4) TDP-43^cyto 5FL^. The TDP-43^cyto^ variants contain point mutations located within the nuclear localization signal (NLS) of TDP-43, resulting in cytoplasmic mislocalization (Igaz et al., 2011; Mann et al., 2019). The 5FL form contains five phenylalanine-to-leucine mutations within the two RNA recognition motif (RRM) domains of TDP-43 that greatly impaired TDP-43’s RNA binding ability and were previously reported to form aggregated “anisomes” inside the nucleus (Cohen et al., 2015; Elden et al., 2010; Mann et al., 2019; Yu et al., 2021). The TDP-43^cyto 5FL^ combines both modifications, leading to insoluble cytoplasmic inclusions (Elden et al., 2010; Keating et al., 2023; Lu et al., 2022; Mann et al., 2019; Yu et al., 2021). Excluding TDP-43^WT^, all three modified versions have proven to sequestrate endogenous TDP-43 into mislocalized or aggregated inclusions (Keating et al., 2023). Therefore, our objective was to utilize CUTS to determine whether the expression of these aggregation-prone TDP-43 variants elicits TDP-43 LOF.

**Figure 3:**
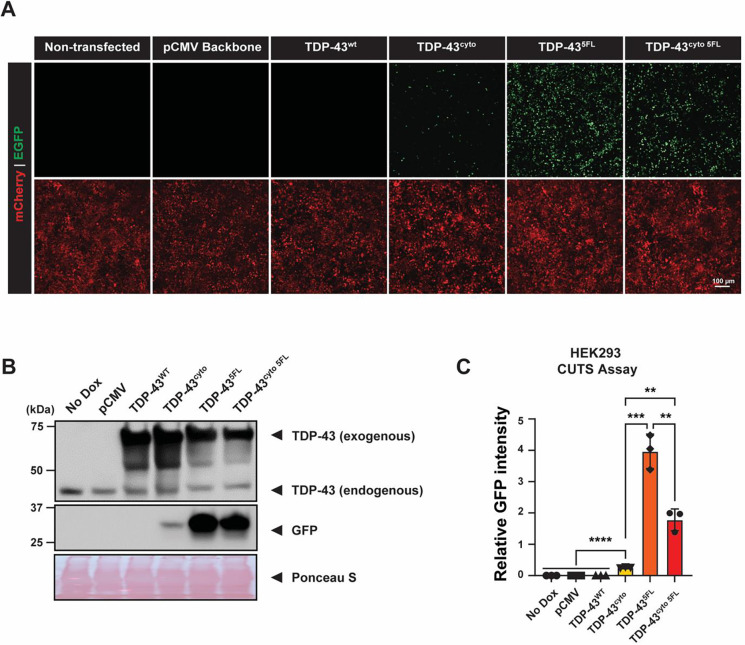
Mislocalization or aberrant phase transitions induce TDP-43 LOF. Stable HEK cells expressing CUTS were induced with doxycycline (1000 ng/mL) for 24 hours before transfection with the following plasmids: pCMV backbone, TDP-43^WT^, TDP-43^ΔNLS^, TDP-43^5FL^, TDP-43^ΔNLS 5FL^, or non-transfected. Following transfection, plasmids were expressed for 72 h, followed by live imaging and protein analysis. **(A)** Live-imaging of CUTS HEK cells expressing WT or mutant TDP-43 gene cassettes. **(B)** Representative western blot of exogenous and endogenous GFP and TDP-43. Ponceau S is shown as a loading control. **(C)** Relative GFP pixel intensity quantification of the band is shown in (B). Statistical significance was determined by one-way ANOVA and Tukey’s multiple comparison test (* = P < 0.03; ** = P < 0.002; *** = P < 0.0002; **** = P < 0.0001). Green = GFP; red = mCherry. Scale bar = 100 μm. N=3 biological replicates.

The introduction of TDP-43^cyto^, TDP-43^5FL^, and TDP-43^cyto 5FL^ induced nuclear GFP signal when assessed by immunofluorescence analysis, while neither the tagged plasmid backbone nor TDP-43^WT^ caused any detectable GFP (Figure 3A). We confirmed the expression of exogenous and endogenous TDP-43 levels by WB and quantified the relative GFP level in each condition (Figure 3B–3C). As we have previously shown that CUTS demonstrates a proportional response to TDP-43’s LOF (Figure 2), we were able to directly interpret the relative ability of the different TDP-43 mutants to trigger TDP-43 LOF by comparison of their GFP levels. All three TDP-43 mutants’ expression triggered significant LOF compared to the control conditions, albeit at varying significance levels. The most modest LOF effect was achieved by TDP-43^cyto^, followed by TDP-43^cyto 5FL^, and TDP-43^5FL^ (Figure 3C). Interestingly, although both TDP-43^5FL^ and TDP-43^cyto 5FL^ caused significantly elevated LOF, the TDP-43^5FL^ mutant alone mediated greater LOF than when combined with the NLS mutations highlighting the potential role of nuclear homotypic TDP-43 interactions potentially contributing to TDP-43 LOF in disease absent its cytoplasmic mislocalization. To further validate the functionality of exogenous TDP-43, we transfected the same tagged TDP-43^WT^ into a HeLa TDP-43 knock-out cell line expressing CUTS (Roczniak Ferguson & Ferguson, 2019). We detected a significantly decreased GFP signal compared to the backbone or non-transfected controls, which confirmed the full splicing function of TDP-43^WT^ (Figure S2A–S2D). Owing to CUTS’ performance, we show the functional consequence caused by TDP-43’s mislocalization and/or aberrant phase transitions, demonstrating that aggregation-prone TDP-43 variants mediated direct LOF toxicity in addition to any gain-of-function (GOF) toxic events. Furthermore, these results strongly support CUTS’s capability in measuring functional TDP-43 levels under broader contexts beyond TDP-43 KD.

### CUTS-mediated autoregulated restoration of TDP-43 splicing.

**Figure 4:**
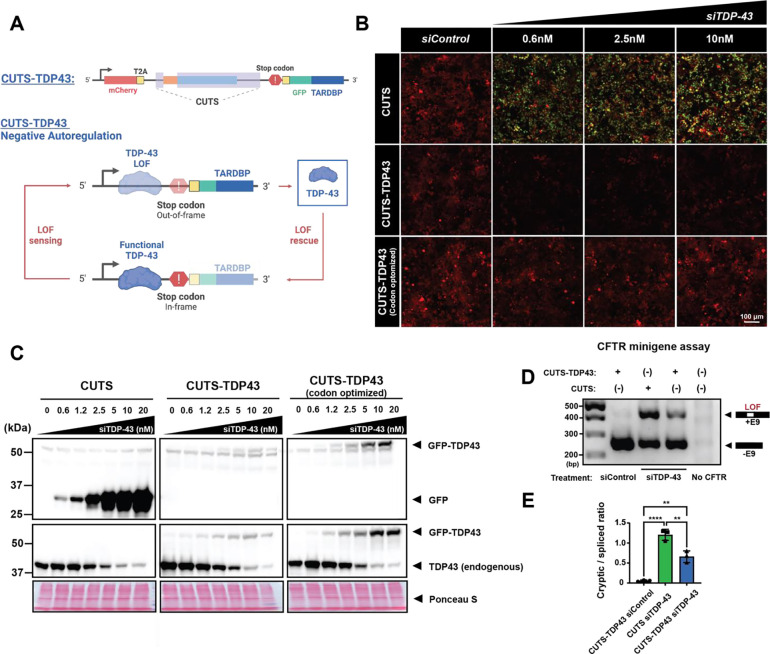
Autoregulatory rescue of TDP-43 loss-of-function by CUTS-TDP43 expression. **(A)** Schematic of CUTS as an autoregulatory controller of TDP-43 expression (CUTS-TDP43). **(B-C)** TDP-43 siRNA (siTDP43) dose-response curve in stable polyclonal HEK cells expressing CUTS, CUTS-TDP43, or CUTS-TDP43 (codon optimized). The codon-optimized variation allows for continued expression during siTDP-43 treatment. HEK cells expressing the CUTS, CUTS-TDP-43 and CUTS-TDP-43 codon optimize system were reverse transfected with control siRNA (siControl) or siTDP43 in a dose-response curve (0.6nM-20nM) in a doxycycline (1000ng/ml) supplement media for 72hr. Cells were then used for live imaging or protein analysis. (B) Live imaging of the CUTS variants. (C) Immunoblot assay of GFP and TDP-43. Ponceau S is shown as a loading control. **(D-E)** CFTR minigene assay in stable CUTS or CUTS-TDP43 (codon optimized) expressing HEK cells. Cells were induced with doxycycline (1000 ng/mL) for 24 h before transfection with the CFTR minigene. Following an additional 24h of expression, cells were transfected with 20nM siControl or siTDP-43. Cells were harvested 48 h following siRNA transfection for RNA extraction and RT-PCR analysis. (D) PCR agarose gel of CFTR minigene. (E) PCR analysis of the ratio between the CFTR cryptic exon inclusion and the correctly spliced product from CFTR as shown in (D). Statistical significance was determined by student t-test (* = P < 0.03; ** = P < 0.002; *** = P < 0.0002; **** = P < 0.0001). Green = GFP; red = mCherry. Scale bar = 100 μm. N=3 biological replicates.

Given the growing recognition of the role TDP-43 LOF is believed to play in disease progression, numerous efforts have been committed to developing rescue methods aimed at re-delivering TDP-43 or other gene payloads to restore its physiological splicing function (Baughn et al., 2023; Mehta et al., 2023; Sun et al., 2017). However, a significant challenge in LOF therapies lies in maintaining precise TDP-43 levels within neurons, as even slight overexpression can lead to GOF toxicity (Johnson et al., 2009; Park et al., 2017; Yang et al., 2022). Consequently, a generalized TDP-43 gene-replacement therapy without genome integration carries a substantial risk of overexpression toxicity. Therefore, a CE biosensor such as CUTS may be used to control cell- and temporal-specific regulation of a gene payload. To test this, we generated a CUTS-controlled TDP-43 (CUTS-TDP43) transgene (Figure 4A). Considering the ultra-sensitivity to TDP-43 LOF and minimal leakage under physiological TDP-43 levels, CUTS-TDP43 may have the potential to autonomously negatively regulate its expression, ensuring levels will not surpass physiological levels.

To test this, we created a new polyclonal stable line in HEK293 cells (CUTS-TDP43) by replacing the 3xNLS in the original CUTS cassette with the *TARDBP* ORF fused to a GFP reporter (Figure 4A). However, as our siTDP43 targets the sequence within the coding region, CUTS-TDP43 was also knocked down upon siRNA transfection, shown by a generalized decreased mCherry signal (Figure 4B). Therefore, we designed a codon-optimized CUTS-TDP43^CO^ that is not targeted by siTDP43 (Figure 4B). Live imaging analyses showed that CUTS GFP signal demonstrated a steady increase in expression in response to increasing doses of siTDP43; however, the GFP signal from CUTS-TDP43^CO^ remained undetectable (Figure 4B). WB analysis further demonstrated successful TDP-43 rescue under endogenous TDP-43 KD, as shown by the increasing exogenous TDP-43 observed following decreases in endogenous TDP-43 (Figure 4C). The amount of total TDP-43 appeared to remain consistent throughout the increasing siTDP43 doses, indicating tight regulation of the rescue parameters. To further confirm whether CUTS-TDP43^CO^ could rescue TDP-43 splicing functionality, we performed a *CFTR* minigene assay (Ayala et al., 2006; Buratti & Baralle, 2001). The expression of CUTS-TDP43^SO^ demonstrated partial, yet significant rescue of cryptic exon 9 splicing in *CFTR* minigene, supporting its controlled efficacy in rescuing splicing LOF (Figure 4D–4E). Taken together, these data indicate that CUTS can autoregulate a TDP-43 payload to physiological levels in response to TDP-43 knockdown.

## Discussion:

We developed and characterized the CUTS system, a novel approach to detect TDP-43 LOF. The CUTS system utilizes TDP-43-dependent CE events to correlate the level of TDP-43 LOF directly with the expression of a reporting gene. By combining the CFTR-TS and UNC13A-TS, our findings demonstrate that the CUTS system provides an optimal balance of sensitivity and accuracy. This was evidenced by the ability of CUTS to detect modest levels of TDP-43 LOF, as shown by the dose-responsive increase in the expression of the reporter gene, GFP, under various siTDP-43 concentrations (Figure 2). Furthermore, our results suggest that CUTS can effectively discern TDP-43 LOF induced by pathological phase transitions or mislocalization, a critical aspect in the context of neurodegenerative diseases containing TDP-43 pathology, such as ALS and FTLD. The CUTS system’s potential for application in gene-replacement therapies was also highlighted, offering a promising avenue for autoregulated rescue of TDP-43 function, which is critical for avoiding the deleterious effects of TDP-43 overexpression.

This tool findings enables for the significant advancement in the capacity to detect TDP-43 LOF using biosensor assays across diverse experimental settings and through multiple analytical methods. Previously, the *CFTR* minigene assay has been the predominant approach for detecting TDP-43 LOF (Ayala et al., 2006; Buratti & Baralle, 2001; Cohen et al., 2015; Conicella et al., 2020; Jiang et al., 2017; Hermann Broder Schmidt et al., 2019). However, this approach is associated with several limitations, all of which are effectively addressed by utilizing the CUTS system. The first advantage of the CUTS system is its ability to detect TDP-43 LOF in real-time through live-imaging analysis. Unlike *CFTR* minigene assays, which typically necessitate endpoint experimental analysis, CUTS facilitates continuous monitoring, eliminating the need for multiple fixed time points. Additionally, CUTS can be seamlessly integrated with various analytical methods, including RT-qPCR (at the RNA level), WB analysis (at the protein level), live imaging (for real-time assessment), and immunofluorescence imaging (to correlate with relevant markers). While not evaluated in this study, it is conceivable that CUTS could be adapted for use with flow cytometry-based techniques, leveraging GFP-positive cells as an output for analysis, as previously demonstrated with a *CFTR*-modified sensor (H Broder Schmidt & Rohatgi, 2020).

In addition to the expanded array of analytical methods offered by CUTS compared to *CFTR* minigene assays, we anticipate that the CUTS system will exhibit superior sensitivity and accuracy. This is supported by the comparison of CUTS with the CFTR-TS or UNC13A-TS cassette (Figure 1), underscoring the potential of CUTS to outperform single minigene-based approaches in TDP-43 LOF detection. While recent work in two recent preprints suggests other CE biosensors are in development, CUTS appears to exhibit enhanced sensitivity (Wilkins et al., 2023; Zhang et al., 2023). The expression of the GFP reporter in CUTS achieved up to 118,224-fold increase upon TDP-43 knockdown, compared to *ADNP2* (< 5-fold) (Zhang et al., 2023); TDP-REGv1 (<20-fold); and TDP-REGv2 (<300-fold) (Wilkins et al., 2023). We also show that CUTS can detect ultra-low levels of TDP-43 knockdown (increasing > 7-fold), below the WB or RT-qPCR detection limit. Furthermore, CUTS exhibits a robust log-linear relationship to siRNA doses, making it suitable for quantitative purposes.

Due to the high flexibility of the CUTS system, its application can be expanded *in vitro* and *in vivo* when coupled with disease models. Integrating the CUTS system with disease models enables the evaluation of the model’s fidelity in recapitulating TDP-43 LOF phenotypes. Such assessments are crucial for selecting appropriate models that faithfully replicate the desired study context. Furthermore, coupling the CUTS system with TDP-43 models presents a valuable approach for diverse screening studies. For example, CUTS can be leveraged for high-throughput drug screening and CRISPR screening methodologies. Such approaches hold promise for uncovering critical insights into cell-specific disease mechanisms, identifying pivotal disease modifiers, and delineating potential therapeutic genetic targets (Aldewachi et al., 2021; Bock et al., 2022).

A significant advantage of the CUTS system lies in its capacity to deliver precisely regulated gene therapy for rescuing TDP-43 LOF. This study illustrates this capability by placing a functional TDP-43 transcript downstream of the CUTS regulatory elements. The system’s self-regulating ability enhances its safety profile as a gene therapy approach, ensuring gene expression occurs only when necessary and exclusively in cells lacking TDP-43 function. Furthermore, this system can be expanded by substituting the TDP-43 transcript with other genetic modifiers of disease, such as antibodies and PROTACs (Pozzi et al., 2019; Tseng et al., 2023), or genes with established therapeutic potential, including heat shock proteins (HSPs) or heterogeneous nuclear ribonucleoproteins (hnRNPs) (Koike et al., 2023; Lu et al., 2022; Yu et al., 2021). This adaptability holds promise for achieving safe therapeutic outcomes without the need for direct TDP-43 expression.

## Methods:

### Generation of plasmids

The CUTS sequence, plasmid, and map were originally generated in this study. The CFTR-TS, UNC13A-TS and CUTS DNA sequences were designed *in silico* and *de novo* synthesized by Genewiz. These sequences were and assembled into Tet3G vector between EcoRI and NotI with NEBuilder HiFi DNA Assembly Master Mix (NEB, E2621L) following the manufacturer’s protocol. The full DNA sequence for CUTS, CFTR-TS, and UNC13A-TS can be found in Table S1.

The CFTR minigene assay plasmid (pTB-CFTR-A455E) was a kind gift from Dr. Yuna Ayala. The exogenous TDP-43 plasmids were constructed in a pCMV backbone by linking a 3xFlag-APEX2 protein (Addgene #164622) (Bonet Ponce et al., 2020) to TDP-43 coding sequences with WT, cyto, 5FL, or cyto 5FL modifications (Mann et al., 2019).

The codon-optimized *TARDBP* coding sequence (Table S1) was synthesized by IDT and assembled downstream of the GFP sequence of CUTS with NEBuilder HiFi DNA Assembly Master Mix.

All the primers were synthesized by IDT. All plasmids were verified using whole-plasmid sequencing via Oxford Nanopore, provided by Plasmidsaurus.

### Cell culture and transfection

Human Embryonic Kidney 293 (HEK293) cells (female genotype, acquired from the American Type Culture Collection (ATCC)) and Hela TDP-43 knock-out (KO) cells (a kind gift from Dr. Shawn M Ferguson) (Roczniak Ferguson & Ferguson, 2019) were cultivated in Dulbecco’s Modified Eagle Medium high glucose, pyruvate (DMEM, Thermo Fisher Scientific, 10–313-039) supplemented with 10% HyClone Bovine Growth Serum (Cytiva HyClon, SH3054103HI) and 1X GlutaMAX (Thermo Fisher Scientific, 10–313-039). Cells were incubated at 37°C in a 5% CO2 atmosphere with high humidity. For transfection assays, cells were plated on collagen-coated coverslips or dishes (50 μg/mL, GIBCO) and transfected with designated DNA quantities using Lipofectamine 3000 (Thermo Scientific, L3000015) following the provider’s protocol.

### HEK293 stable cell line generation via Piggybac transposition

For stable cell line creation, HEK293 cells were pre-plated on 6-well plates and transfected at approximately 70% confluence with 2.5 μg of Piggybac plasmids encoding CUTS, CFTR-TS, UNC13A-TS, and CUTS-TDP43 alongside 0.5 μg of the Super PiggyBac Transposase Expressing plasmid (PB200PA-1) using Lipofectamine 3000, according to the manufacturer’s guidelines. A transfection control without transposase was included. Following a 48-hour post-transfection period, cells were selected with puromycin (Sigma, P8833) at 5 μg/mL, with media changes every two days. Selection resulted in control cell death within approximately 5 days, while surviving populations were expanded and maintained in reduced puromycin concentrations (2.5 μg/mL) to establish stable lines. Expression of the transgenes was confirmed by immunofluorescence staining and Western blot analysis.

### SDS-PAGE and Western blot

For protein analysis, cells were lysed directly on the plate using fresh and pre-chilled Urea-RIPA buffer: 2M fresh urea in 1XRIPA buffer (Boston Bioproducts, BP-115X), supplemented with 1% protease inhibitor cocktail (Sigma, P8340) and sonicated. Protein concentrations were quantified using the Pierce BCA Protein Assay Kit (Thermo Scientific, 23227). Proteins were resolved by SDS-PAGE and transferred to nitrocellulose membranes for WB analysis. Membranes were blocked and probed with primary antibodies: mouse-anti-GFP (Santa Cruz, sc-9996, 1:200), mouse-anti-α-tubulin (Sigma, T5168, 1:1000), rabbit-anti-TDP-43 (Proteintech, 10782–2-AP, 1:2500), and rabbit-anti-mCherry (Cell Signaling, 43590, 1:1000), followed by HRP-conjugated secondary antibodies: donkey-anti-mouse (JacksonImmunoResearch 715035151, 1:5000) or donkey-anti-rabbit (JacksonImmunoResearch 711035152, 1:5000). Detection was achieved using Western Lightning ECL Pro (Revvity, NEL1201001EA) or Supersignal West Femto Maximum Sensitivity Chemiluminescent Substrate (Thermo Scientific, 34095) in an Amersham ImageQuant 800 GxP biomolecular imager system (Amersham, 29653452).

### Confocal microscopy

Confocal imaging was performed on a Nikon A1 laser-scanning microscope using either a 60X oil immersion or a 10X/20X objective for live-cell observations. A Tokai HIT stage-top incubator maintained the required environmental conditions. Nikon Elements software facilitated image acquisition and analysis. Representative images were chosen from at least two independent experiments with a minimum of three biological replicates each.

### siRNA reverse transfection

siRNA reverse transfections were conducted using Lipofectamine RNAiMAX reagent (Thermo Scientific, 13778150), adhering to the supplier’s protocol. To knockdown TDP-43, the following siRNAs were used: ON-TARGETplus SMARTpool siRNA against *TARDBP* (Dharmaco, L-012394–00-0005) and siGENOME non-Targeting siRNA for control (Dharmaco, D-001206–13-05).

### RNA extraction, RT-PCR, and qPCR

RNA extraction was performed using the RNeasy Mini Kit (Qiagen, 74106) with concentration determinations via Nanodrop Spectrophotometer (Nanodrop, ND-1000). Reverse transcription of extracted RNA (0.2 μg) to cDNA utilized iScript Reverse Transcription Supermix (Bio-Rad, 1708841) in accordance with the manufacturer’s guidelines.

The RT-PCR or RT-qPCR was conducted with cDNA diluted 10-fold. For RT-PCR assay, CFTR cryptic exon region was amplified with the following primer pair: P690-F (5’-CAACTTCAAGCTCCTAAGCCACTGCCTGC) and P691-R (5’-TAGGATCCGGTCACCAGGAAGTTGGTTAAATCA). CUTS’ cryptic exon region was amplified with the following primer pair: CUTS-CE-F (5’-ATCCCGGCCCTGGATCCG) and CUTS-CE-R (5’-GTCAGCTTGCCGTAGGTGGC). PCR products were separated by agarose gel electrophoresis, and the bands were visualized with Amersham ImageQuant 800 GxP biomolecular imager system.

For RT-qPCR assay, SsoAdvanced^™^ Universal SYBR Green Supermix (Biorad, 1725272) was used following the supplier’s protocol on a CFX96 Touch Real-Time PCR Detection System (Biorad). Three technical replicates were included for each sample with the following program: 95°C for 30 s, 40 cycles of 95°C for 15 s and 60°C for 20 s. CUTS-CE-F (5’-ATCCCGGCCCTGGATCCG) and CUTS-CE-R (5’-GTCAGCTTGCCGTAGGTGGC) were used to quantify normal CUTS transcript. CUTS-J-F (5’-TCCGGCGAGGGATTTGGG) and CUTS-J-R (5’-CCCCACCTAGACCCATCTCTCC) were primers targeting the cryptic exon junctions to quantify cryptic exon-specific CUTS transcript. Relative quantification of CUTS cryptic exon was determined by the ΔCt value of CUTS-J normalized to CUTS-CE.

### Statistical analysis

Statistical significance was evaluated using GraphPad Prism 9 software, and specific tests used for each experiment are outlined in the respective figure legends.

## Supplementary Material

Supplement 1

## Highlights

- CUTS is a cryptic exon RNA biosensor enabling real-time detection of TDP-43 loss of splicing function.
- CUTS exhibits a significant linear relationship with TDP-43 loss.
- CUTS can deliver an autoregulated gene payload in response to TDP-43 loss-of-function.
- TDP-43 homotypic phase transitions induce loss of splicing function via CUTS detection.
