# Supplementary material for: CUTS RNA Biosensor for the Real-Time Detection of TDP-43 Loss-of-Function
Source: bioRxiv. 2024 Jul 12:2024.07.12.603231. Preprint. [Version 1] doi: 10.1101/2024.07.12.603231 (PMC11257528; doi:10.1101/2024.07.12.603231)
Supplement: Supplement 1 [file NIHPP2024.07.12.603231v1-supplement-1.pdf]

bioRxiv preprint doi: https://doi.org/10.1101/2024.07.12.603231; this version posted July 12, 2024. The copyright holder for this preprint (which was not certified by peer review) is the author/funder, who has granted bioRxiv a license to display the preprint in perpetuity. It is made available under aCC-BY 4.0 International license.

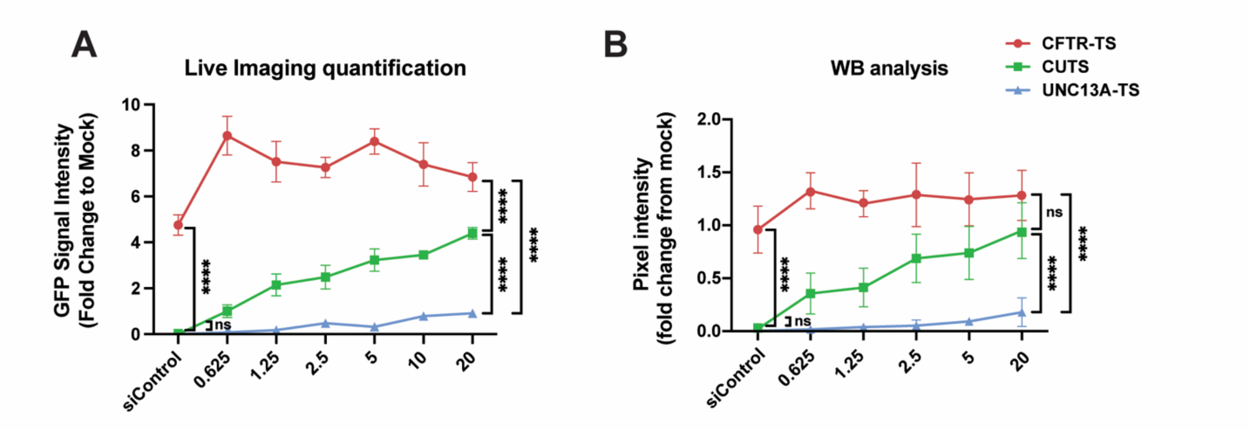

Supplementary Figure 1: Dose-response of CUTS and TDP-43 knockdown. *Comparison of stable polyclonal HEK cells expressing UNC13A-TS, CFTR-TS, or CUTS following treatment with siRNA control (siControl) (20nM) or TDP-43 (siTDP-43) (0.6nM - 20nM). Cells were reverse transfected with siRNA treatment in complete media supplemented with doxycycline (1000 ng/mL). After 72 h, cells were analyzed by live imagining and protein lysate was harvested for western blot analysis. (**A**) Mean intensity quantification of GFP signal intensity from live imaging, presented as fold change from mock.* ***(B)*** *Relative pixel quantification of GFP normalized to total protein (Ponceau S), presented as fold change from mock. Statistical significance was determined by two-way ANOVA and Tukey's multiple comparison test (* = P < 0.03; ** = P < 0.002; *** = P < 0.0002; **** = P < 0.0001). N=3 biological replicates.*

bioRxiv preprint doi: https://doi.org/10.1101/2024.07.12.603231; this version posted July 12, 2024. The copyright holder for this preprint (which was not certified by peer review) is the author/funder, who has granted bioRxiv a license to display the preprint in perpetuity. It is made available under aCC-BY 4.0 International license.

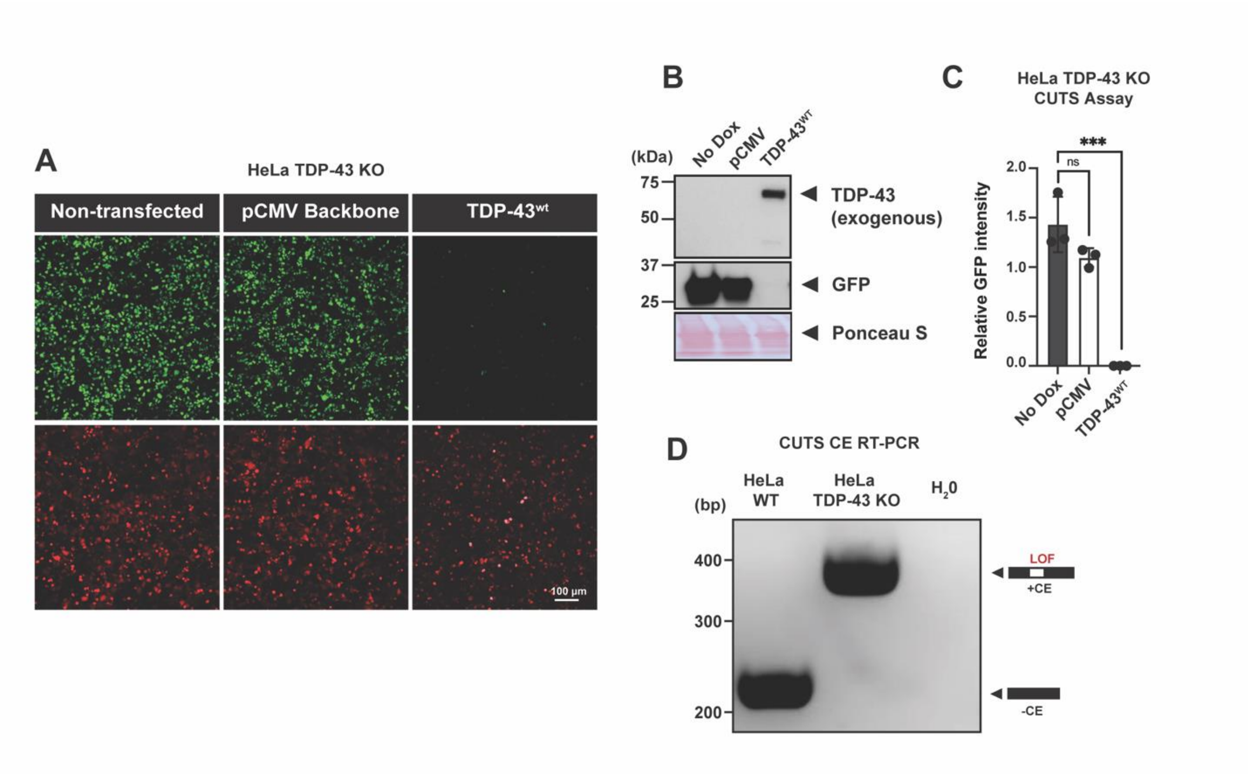

Supplementary Figure 2: TDP-43^{wt} expression rescues loss-of-function in a HeLa TARDBP Knockout Cell Line. *Transient CUTS expression in Hela TDP-43 KO we induced with doxocycline (1000n/ml) for 24 hours before transfection of pCM backbone or TDP-43^{WT} plasmids. Following transfection, plasmids were expressed for 72 h, followed by live imaging and protein analysis.* ***(A)*** *Live-imaging of Hela TDP-43 KO expressing CUTS in combination with TDP-43^{WT} or pCMV backbone control.* ***(B)*** *Representative WB of exogenous and endogenous GFP and TDP-43. Ponceau S is shown as a loading control.* ***(C)*** *Relative GFP pixel intensity quantification of the protein bands shown in (B). (**D**) Agarose gel showing RT-PCR product from CUTS cryptic exon inclusion from wildtype or TDP-43 KO Hela cell lines using the CUTS-CE primers shown in Figure 2E. Statistical significance was determined by one-way ANOVA and*

bioRxiv preprint doi: https://doi.org/10.1101/2024.07.12.603231; this version posted July 12, 2024. The copyright holder for this preprint (which was not certified by peer review) is the author/funder, who has granted bioRxiv a license to display the preprint in perpetuity. It is made available under aCC-BY 4.0 International license.

Tukey's multiple comparison test (* = $P < 0.03$; ** = $P < 0.002$; *** = $P < 0.0002$; **** = $P < 0.0001$). Green = GFP; red = mCherry. Scale bar = 100 µm. N=3 biological replicates.

bioRxiv preprint doi: https://doi.org/10.1101/2024.07.12.603231; this version posted July 12, 2024. The copyright holder for this preprint (which was not certified by peer review) is the author/funder, who has granted bioRxiv a license to display the preprint in perpetuity. It is made available under aCC-BY 4.0 International license.

Supplementary Table 1. Information of DNA sequence

**Domains of the sequence**	**Sequence**
**1. UNC13A-TS**<br>mCherry<br>Linker-T2A<br>HBA1_exon2<br>HBA1_intron 2<br>HBA1_exon3<br>UNC13A_modified_CE<br>UNC13A_intron20<br>Linker-T2A<br>EGFP<br>3XNLS	ATGGTGAGCAAGGGCGAGGAGGATAACATGGCCATCATCAAGG<br>AGTTCATGCGCTTCAAGGTGCACATGGAGGGCTCCGTGAACGG<br>CCACGAGTTCGAGATCGAGGGCGAGGGCGAGGGCCGCCCCTA<br>CGAGGGCACCCAGACCGCCAAGCTGAAGGTGACCAAGGGTGG<br>CCCCCTGCCCTTCGCCTGGGACATCCTGTCCCCTCAGTTCATGT<br>ACGGCTCCAAGGCCTACGTGAAGCACCCCGCCGACATCCCCGA<br>CTACTTGAAGCTGTCCTTCCCCGAGGGCTTCAAGTGGGAGCGC<br>GTGATGAACTTCGAGGACGGCGGCGTGGTGACCGTGACCCAGG<br>ACTCCTCCCTGCAGGACGGCGAGTTCATCTACAAGGTGAAGCTG<br>CGCGGCACCAACTTCCCCTCCGACGGCCCCGTAATGCAGAAGA<br>AGACCATGGGCTGGGAGGCCTCCTCCGAGCGGATGTACCCCGA<br>GGACGGCGCCCTGAAGGGCGAGATCAAGCAGAGGCTGAAGCT<br>GAAGGACGGCGGCCACTACGACGCTGAGGTCAAGACCACCTAC<br>AAGGCCAAGAAGCCCGTGCAGCTGCCCGGCGCCTACAACGTCA<br>ACATCAAGTTGGACATCACCTCCCACAACGAGGACTACACCATC<br>GTGGAACAGTACGAACGCGCCGAGGGCCGCCACTCCACCGGC<br>GGCATGGACGAGCTGTACAAGGGAGGTGGGGGATCTGGTGAG<br>GGCAGAGGCAGTCTGCTGACATGCGGTGACGTGGAAGAGAATC<br>CCGGCCCTGGATCCGGCCCGGTCAACTTCAAGGTGAGCGGCGG<br>GCCGGGAGCGATCTGGGTCGAGGGGCGAGATGGCGCCTTCCT<br>CGCAGGGCAGAGGATCACGCGGGTTGCGGGAGGTGTAGCGCA<br>GGCGGCGGCTGCGGGCCTGGGCCCTCGGCCCCACTGACCCTC<br>TTCTCTGCACAGCTCCTAAGCCACTGCCTGCTGGTGACCTCTGC<br>CTGGGTTTCCTGGAAAGAACTCTTATCCCCAGGAACTAGTTTGTT<br>GAATAAATGCTGGTGTGAATGTGAATGGATGATTGAACAGATGA<br>ATGAGTGTGATGAGTATGATAAAAGCATGGATGGAGAGATGGGT<br>GAGTACATGGATGGATAGATGGATGAGTTGGTGGGTAGATTCGT<br>GGCTAGATGGATGATGGATGGATGGATGGATGGATGGAT<br>GGACAGATGGATGGATATATGATTGAACTATTGAAAGTATAGATG<br>TATGGATGGGTGAATTTGGGGGTAATTGTTAGATGATGGATGTG<br>GATGGTTGTGAGTGGCTGGTGGACAGACGAAAAATGGATGGGT<br>GGATAAATTGATGGGTGGATGGATGGTTGGTTGTATGAAAGAAT<br>GAATGATTGGGTAGATAAAAGAGTAGATGAATGAATTAATGAATA<br>AACAGGCAGATGGATGATGTAAGCTGCCCCAGACCCTGGGACC<br>TCTGACCCCCGGCGACCCCTTGCACTCTCCATGAGCCCTTTCCT

bioRxiv preprint doi: https://doi.org/10.1101/2024.07.12.603231; this version posted July 12, 2024. The copyright holder for this preprint (which was not certified by peer review) is the author/funder, who has granted bioRxiv a license to display the preprint in perpetuity. It is made available under aCC-BY 4.0 International license.

	TTTTTTTCCTCAGGTCTAGGTGGGGGATCTGGTGAGGGCAGAGG<br>CAGTCTGCTGACATGCGGTGACGTGGAAGAGAATCCCGGCCCT<br>TCTAGAAGCAAGGGCGAGGAGCTGTTCACCGGGGTGGTGCCCA<br>TCCTGGTCGAGCTGGACGGCGACGTAAACGGCCACAAGTTCAG<br>CGTGTCCGGCGAGGGCGAGGGCGATGCCACCTACGGCAAGCT<br>GACCCTGAAGTTCATCTGCACCACCGGCAAGCTGCCCGTGCCC<br>TGGCCCACCCTCGTGACCACCCTGACCTACGGCGTGCAGTGCT<br>TCAGCCGCTACCCCGACCACATGAAGCAGCACGACTTCTTCAAG<br>TCCGCCATGCCCGAAGGCTACGTCCAGGAGCGCACCATCTTCTT<br>CAAGGACGACGGCAACTACAAGACCCGCGCCGAGGTGAAGTTC<br>GAGGGCGACACCCTGGTGAACCGCATCGAGCTGAAGGGCATCG<br>ACTTCAAGGAGGACGGCAACATCCTGGGGCACAAGCTGGAGTA<br>CAACTACAACAGCCACAACGTCTATATCATGGCCGACAAGCAGA<br>AGAACGGCATCAAGGTGAACTTCAAGATCCGCCACAACATCGAG<br>GACGGCAGCGTGCAGCTCGCCGACCACTACCAGCAGAACACCC<br>CCATCGGCGACGGCCCCGTGCTGCTGCCCGACAACCACTACCT<br>GAGCACCCAGTCCGCCCTGAGCAAAGACCCCAACGAGAAGCGC<br>GATCACATGGTCCTGCTGGAGTTCGTGACCGCCGCCGGGATCA<br>CTCTCGGCATGGACGAGCTGTACAAGCTGCAGAGCAGGGCCGA<br>CCCCAAGAAGAAGAGGAAGGTGGACCCCAAGAAGAAGAGGAAG<br>GTGGACCCCAAGAAGAAGAGGAAGGTGTGA
**2. CFTR-TS**<br>mCherry<br>Linker-T2A<br>CFTR_intron 8<br>CFTR_modified_exon9<br>CFTR_intron 9<br>Linker-T2A<br>EGFP<br>3XNLS	ATGGTGAGCAAGGGCGAGGAGGATAACATGGCCATCATCAAGG<br>AGTTCATGCGCTTCAAGGTGCACATGGAGGGCTCCGTGAACGG<br>CCACGAGTTCGAGATCGAGGGCGAGGGCGAGGGCCGCCCCTA<br>CGAGGGCACCCAGACCGCCAAGCTGAAGGTGACCAAGGGTGG<br>CCCCCTGCCCTTCGCCTGGGACATCCTGTCCCCTCAGTTCATGT<br>ACGGCTCCAAGGCCTACGTGAAGCACCCCGCCGACATCCCCGA<br>CTACTTGAAGCTGTCCTTCCCCGAGGGCTTCAAGTGGGAGCGC<br>GTGATGAACTTCGAGGACGGCGGCGTGGTGACCGTGACCCAGG<br>ACTCCTCCCTGCAGGACGGCGAGTTCATCTACAAGGTGAAGCTG<br>CGCGGCACCAACTTCCCCTCCGACGGCCCCGTAATGCAGAAGA<br>AGACCATGGGCTGGGAGGCCTCCTCCGAGCGGATGTACCCCGA<br>GGACGGCGCCCTGAAGGGCGAGATCAAGCAGAGGCTGAAGCT<br>GAAGGACGGCGGCCACTACGACGCTGAGGTCAAGACCACCTAC<br>AAGGCCAAGAAGCCCGTGCAGCTGCCCGGCGCCTACAACGTCA<br>ACATCAAGTTGGACATCACCTCCCACAACGAGGACTACACCATC<br>GTGGAACAGTACGAACGCGCCGAGGGCCGCCACTCCACCGGC<br>GGCATGGACGAGCTGTACAAGGGAGGTGGGGGATCTGGTGAG<br>GGCAGAGGCAGTCTGCTGACATGCGGTGACGTGGAAGAGAATC

bioRxiv preprint doi: https://doi.org/10.1101/2024.07.12.603231; this version posted July 12, 2024. The copyright holder for this preprint (which was not certified by peer review) is the author/funder, who has granted bioRxiv a license to display the preprint in perpetuity. It is made available under aCC-BY 4.0 International license.

	CCGGCCCTGGATCCGGCGAGGTGAGTATGGTACATAAAACAAG<br>CATCTATTGAAAATATCTGACAAACTCATCTTTTATTTTTGATGTG<br>TGTGTGTGTGTGTGTGTGTGTGTTTTTTTTTAACAGGGATTTGGG<br>GAATTATTTGAGAAAGCAAAACAAAACAATAACAATAGAAAAACT<br>TCTAATGGTGATGACAGCCAAGATAGAAAGAGGACAGTTGTTGG<br>AGGTTGCTGGATCCACTGGAGCAGGCAAGGTAGTTCTTTTGTTC<br>TTCACTATTAAGAACTTAATTTGGTGCCCATGTCTCTTTTTTTTTC<br>TAGTTTGTAGTGCTGGAAGGTATTTTTGGAGAAATTCTTACATGA<br>GCATTAGGAGAATGTATGGGTGTAGTGTCTTGTATAATAGAAATT<br>GTTCCACTGATAATTTACTCTAGTTTTTTATTTCCTCATATTATTTT<br>CAGTGGCTTTTTCTTCCACATCTTTATATTTTGCACCACATTCAAC<br>ACTGTATCTTGCACATGGCGAGCATATGGTCGATGATGTAAGCT<br>GCCCCAGACCCTGGGACCTCTGACCCCCGGCGACCCCTTGCAC<br>TCTCCATGAGCCCTTTCCTTTTTTTTTCCTCAGGTCTAGGTGGGGG<br>ATCTGGTGAGGGCAGAGGCAGTCTGCTGACATGCGGTGACGTG<br>GAAGAGAATCCCGGCCCTTCTAGAAGCAAGGGCGAGGAGCTGT<br>TCACCGGGGTGGTGCCCATCCTGGTCGAGCTGGACGGCGACGT<br>AAACGGCCACAAGTTCAGCGTGTCCGGCGAGGGCGAGGGCGAT<br>GCCACCTACGGCAAGCTGACCCTGAAGTTCATCTGCACCACCG<br>GCAAGCTGCCCGTGCCCTGGCCCACCCTCGTGACCACCCTGAC<br>CTACGGCGTGCAGTGCTTCAGCCGCTACCCCGACCACATGAAG<br>CAGCACGACTTCTTCAAGTCCGCCATGCCCGAAGGCTACGTCCA<br>GGAGCGCACCATCTTCTTCAAGGACGACGGCAACTACAAGACCC<br>GCGCCGAGGTGAAGTTCGAGGGCGACACCCTGGTGAACCGCAT<br>CGAGCTGAAGGGCATCGACTTCAAGGAGGACGGCAACATCCTG<br>GGGCACAAGCTGGAGTACAACTACAACAGCCACAACGTCTATAT<br>CATGGCCGACAAGCAGAAGAACGGCATCAAGGTGAACTTCAAGA<br>TCCGCCACAACATCGAGGACGGCAGCGTGCAGCTCGCCGACCA<br>CTACCAGCAGAACACCCCCATCGGCGACGGCCCCGTGCTGCTG<br>CCCGACAACCACTACCTGAGCACCCAGTCCGCCCTGAGCAAAG<br>ACCCCAACGAGAAGCGCGATCACATGGTCCTGCTGGAGTTCGT<br>GACCGCCGCCGGGATCACTCTCGGCATGGACGAGCTGTACAAG<br>CTGCAGAGCAGGGCCGACCCCAAGAAGAAGAGGAAGGTGGACC<br>CCAAGAAGAAGAGGAAGGTGGACCCCAAGAAGAAGAGGAAGGT<br>GTGA
**3. CUTS**<br>mCherry<br>Linker-T2A	ATGGTGAGCAAGGGCGAGGAGGATAACATGGCCATCATCAAGG<br>AGTTCATGCGCTTCAAGGTGCACATGGAGGGCTCCGTGAACGG<br>CCACGAGTTCGAGATCGAGGGCGAGGGCGAGGGCCGCCCCTA<br>CGAGGGCACCCAGACCGCCAAGCTGAAGGTGACCAAGGGTGG

bioRxiv preprint doi: https://doi.org/10.1101/2024.07.12.603231; this version posted July 12, 2024. The copyright holder for this preprint (which was not certified by peer review) is the author/funder, who has granted bioRxiv a license to display the preprint in perpetuity. It is made available under aCC-BY 4.0 International license.

CFTR_intron 8<br>CFTR_modified_exon9<br>UNC13A_modified_CE<br>UNC13A_intron20<br>Linker-T2A<br>EGFP<br>3XNLS	CCCCCTGCCCTTCGCCTGGGACATCCTGTCCCCTCAGTTCATGT<br>ACGGCTCCAAGGCCTACGTGAAGCACCCCGCCGACATCCCCGA<br>CTACTTGAAGCTGTCCTTCCCCGAGGGCTTCAAGTGGGAGCGC<br>GTGATGAACTTCGAGGACGGCGGCGTGGTGACCGTGACCCAGG<br>ACTCCTCCCTGCAGGACGGCGAGTTCATCTACAAGGTGAAGCTG<br>CGCGGCACCAACTTCCCCTCCGACGGCCCCGTAATGCAGAAGA<br>AGACCATGGGCTGGGAGGCCTCCTCCGAGCGGATGTACCCCGA<br>GGACGGCGCCCTGAAGGGCGAGATCAAGCAGAGGCTGAAGCT<br>GAAGGACGGCGGCCACTACGACGCTGAGGTCAAGACCACCTAC<br>AAGGCCAAGAAGCCCGTGCAGCTGCCCGGCGCCTACAACGTCA<br>ACATCAAGTTGGACATCACCTCCCACAACGAGGACTACACCATC<br>GTGGAACAGTACGAACGCGCCGAGGGCCGCCACTCCACCGGC<br>GGCATGGACGAGCTGTACAAGGGAGGTGGGGGATCTGGTGAG<br>GGCAGAGGCAGTCTGCTGACATGCGGTGACGTGGAAGAGAATC<br>CCGGCCCTGGATCCGGCGAGGTGAGTATGGTACATAAAACAAG<br>CATCTATTGAAAATATCTGACAAACTCATCTTTTATTTTTGATGTG<br>TGTGTGTGTGTGTGTGTGTGTGTTTTTTTTTAACAGGGATTTGGG<br>GAATTATTTGAGAAAGCAAAACAAAACAATAACAATAGAAAAACT<br>TCTAATGGTGATGACAGCACTGCCTGGGTTTCCTGGAAAGAACT<br>CTTATCCCCAGGAACTAGTTTGTTGAATAAATGCTGGTGTGAATG<br>TGAATGGATGATTGAACAGATGAATGAGTGTGATGAGTATGATAA<br>AAGCATGGATGGAGAGATGGGTGAGTACATGGATGGATAGATG<br>GATGAGTTGGTGGGTAGATTCGTGGCTAGATGGATGATGGATGG<br>ATGGATGGATGGATGGATGGACAGATGGATGGATATATGA<br>TTGAACTATTGAAAGTATAGATGTATGGATGGGTGAATTTGGGG<br>GTAATTGTTAGATGATGGATGTGGATGGTTGTGAGTGGCTGGTG<br>GACAGACGAAAAATGGATGGGTGGATAAATTGATGGGTGGATGG<br>ATGGTTGGTTGTATGAAAGAATGAATGATTGGGTAGATAAAAGAG<br>TAGATGAATGAATTAATGAATAAACAGGCAGATGGATGATGTAAG<br>CTGCCCCAGACCCTGGGACCTCTGACCCCCGGCGACCCCTTGC<br>ACTCTCCATGAGCCCTTTCCTTTTTTTTCCTCAGGTCTAGGTGGG<br>GGATCTGGTGAGGGCAGAGGCAGTCTGCTGACATGCGGTGACG<br>TGGAAGAGAATCCCGGCCCTTCTAGAAGCAAGGGCGAGGAGCT<br>GTTCACCGGGGTGGTGCCCATCCTGGTCGAGCTGGACGGCGAC<br>GTAAACGGCCACAAGTTCAGCGTGTCCGGCGAGGGCGAGGGC<br>GATGCCACCTACGGCAAGCTGACCCTGAAGTTCATCTGCACCAC<br>CGGCAAGCTGCCCGTGCCCTGGCCCACCCTCGTGACCACCCTG<br>ACCTACGGCGTGCAGTGCTTCAGCCGCTACCCCGACCACATGA<br>AGCAGCACGACTTCTTCAAGTCCGCCATGCCCGAAGGCTACGTC<br>CAGGAGCGCACCATCTTCTTCAAGGACGACGGCAACTACAAGAC

bioRxiv preprint doi: https://doi.org/10.1101/2024.07.12.603231; this version posted July 12, 2024. The copyright holder for this preprint (which was not certified by peer review) is the author/funder, who has granted bioRxiv a license to display the preprint in perpetuity. It is made available under aCC-BY 4.0 International license.

	CCGCGCCGAGGTGAAGTTCGAGGGCGACACCCTGGTGAACCGC<br>ATCGAGCTGAAGGGCATCGACTTCAAGGAGGACGGCAACATCC<br>TGGGGCACAAGCTGGAGTACAACTACAACAGCCACAACGTCTAT<br>ATCATGGCCGACAAGCAGAAGAACGGCATCAAGGTGAACTTCAA<br>GATCCGCCACAACATCGAGGACGGCAGCGTGCAGCTCGCCGAC<br>CACTACCAGCAGAACACCCCCATCGGCGACGGCCCCGTGCTGC<br>TGCCCGACAACCACTACCTGAGCACCCAGTCCGCCCTGAGCAA<br>AGACCCCAACGAGAAGCGCGATCACATGGTCCTGCTGGAGTTC<br>GTGACCGCCGCCGGGATCACTCTCGGCATGGACGAGCTGTACA<br>AGCTGCAGAGCAGGGCCGACCCCAAGAAGAAGAGGAAGGTGGA<br>CCCCAAGAAGAAGAGGAAGGTGGACCCCAAGAAGAAGAGGAAG<br>GTGTGA
**4. Codon-optimized TDP-43**	ATGTCTGAATATATTCGGGTAACCGAAGATGAGAACGATGAACC<br>AATCGAAATTCCAAGTGAAGACGACGGAACAGTCTTGCTCTCCA<br>CAGTAACGGCGCAGTTTCCCGGTGCGTGCGGATTGAGATATCG<br>CAACCCGGTGTCTCAGTGCATGCGAGGTGTAAGGTTGGTCGAA<br>GGGATCCTGCACGCCCCCGATGCAGGCTGGGGTAACCTCGTGT<br>ACGTGGTTAACTACCCGAAGGATAACAAGAGGAAAATGGATGAA<br>ACCGATGCTAGTTCCGCCGTGAAGGTGAAACGAGCGGTTCAAAA<br>AACTTCAGATCTTATAGTTCTGGGCCTCCCTTGGAAGACCACGG<br>AACAGGATTTGAAAGAGTATTTTTCAACCTTTGGCGAGGTTCTCA<br>TGGTGCAGGTTAAAAAAGATCTTAAAACCGGACACTCAAAAGGA<br>TTTGGCTTCGTGAGGTTCACCGAGTACGAAACGCAGGTAAAAGT<br>GATGTCACAGCGCCATATGATAGACGGACGGTGGTGCGATTGTA<br>AGCTGCCCAATTCCAAGCAAAGCCAAGACGAGCCTCTGAGGAGT<br>CGCAAAGTTTTCGTTGGACGCTGTACGGAAGACATGACTGAAGA<br>TGAGCTTAGGGAGTTTTTTAGTCAGTATGGAGACGTTATGGATGT<br>CTTTATCCCAAAACCGTTTCGGGCCTTCGCCTTTGTCACCTTTGC<br>CGACGACCAGATCGCCCAGAGCTTGTGTGGCGAAGACCTGATA<br>ATTAAAGGTATATCTGTCCATATCAGCAATGCGGAACCGAAACAC<br>AACTCTAATCGACAGCTTGAGAGGTCAGGGAGGTTTGGGGGTAA<br>CCCGGGTGGATTCGGTAACCAAGGGGGGTTCGGAAACTCCCGG<br>GGCGGAGGAGCCGGATTGGGTAATAACCAGGGATCCAACATGG<br>GCGGCGGGATGAACTTTGGTGCCTTCTCCATAAATCCTGCGATG<br>ATGGCCGCAGCGCAAGCTGCCCTTCAGAGCTCCTGGGGTATGA<br>TGGGGATGCTTGCTTCTCAGCAAAACCAATCCGGGCCCAGCGG<br>CAACAATCAGAATCAAGGTAATATGCAGCGAGAACCGAACCAGG<br>CCTTCGGAAGCGGGAATAATTCCTACTCAGGATCAAATAGTGGT<br>GCAGCCATTGGCTGGGGCTCAGCGTCTAATGCAGGCTCAGGCA

bioRxiv preprint doi: https://doi.org/10.1101/2024.07.12.603231; this version posted July 12, 2024. The copyright holder for this preprint (which was not certified by peer review) is the author/funder, who has granted bioRxiv a license to display the preprint in perpetuity. It is made available under aCC-BY 4.0 International license.

	GTGGGTTTAATGGGGGCTTTGGGAGCTCCATGGACAGCAAGTC ATCAGGCTGGGGGTAA
